# Use of Onion Waste as Fuel for the Generation of Bioelectricity

**DOI:** 10.3390/molecules27030625

**Published:** 2022-01-19

**Authors:** Rojas-Flores Segundo, Magaly De La Cruz-Noriega, Nélida Milly Otiniano, Santiago M. Benites, Mario Esparza, Renny Nazario-Naveda

**Affiliations:** 1 Instituto de Investigación en Ciencias y Tecnología de la Universidad Cesar Vallejo, Trujillo 13001, Peru; mdelacruzn@ucv.edu.pe (M.D.L.C.-N.); notiniano@ucv.edu.pe (N.M.O.); 2 Vicerrectorado de Investigación, Universidad Autónoma del Perú, Lima 15842, Peru; santiago.benites@autonoma.pe; 3 Laboratorio Generbim (Genetica, Reproduccion y Biologia Molecular), Escuela de Medicina Humana, Facultad de Medicina Humana, Universidad Privada Antenor Orrego, Trujillo 13001, Peru; mrodrigount@yahoo.com; 4 Grupo de Investigación en Ciencias Aplicadas y Nuevas Tecnologías, Universidad Privada del Norte, Trujillo 13007, Peru; renny.nazario@upn.edu.pe

**Keywords:** organic waste, generation, electricity, onion, microbial fuel cells

## Abstract

The enormous environmental problems that arise from organic waste have increased due to the significant population increase worldwide. Microbial fuel cells provide a novel solution for the use of waste as fuel for electricity generation. In this investigation, onion waste was used, and managed to generate maximum peaks of 4.459 ± 0.0608 mA and 0.991 ± 0.02 V of current and voltage, respectively. The conductivity values increased rapidly to 179,987 ± 2859 mS/cm, while the optimal pH in which the most significant current was generated was 6968 ± 0.286, and the ° Brix values decreased rapidly due to the degradation of organic matter. The microbial fuel cells showed a low internal resistance (154,389 ± 5228 Ω), with a power density of 595.69 ± 15.05 mW/cm^2^ at a current density of 6.02 A/cm^2^; these values are higher than those reported by other authors in the literature. The diffractogram spectra of the onion debris from FTIR show a decrease in the most intense peaks, compared to the initial ones with the final ones. It was possible to identify the species *Pseudomona eruginosa*, *Acinetobacter bereziniae*, *Stenotrophomonas maltophilia*, and *Yarrowia lipolytica* adhered to the anode electrode at the end of the monitoring using the molecular technique.

## 1. Introduction

In recent decades, the rapid growth of human society has brought with it an increase in pollution, in which organic waste due to the lack of solid waste collection centers is dumped around supply centers [1,2]. This situation causes discomfort to homes near these markets due to bad smells, as well as birds and rodents originating from the extensive duration of accumulation [3]. According to the World Bank, people will produce 2.2 trillion tons of solid waste in 2025 [4], of which approximately 33% will be waste that will not be managed safely [5]. According to Szulc et al. (2021), each person produces an average of 0.74 kg per day, but it can vary from 0.11 to 4.54 kg due to people’s diet [6]. Considering that some regions are developing rapidly, this will double or triple their waste production [7]. Due to the large amounts of waste, many researchers have looked for innovative ways to use it; for example, waste has been used in fertilizers [8], biopolymers [9], biogas generation [10], and bioelectricity [11]. In this sense, the use of organic waste to generate electricity through microbial fuel cells is being intensively investigated, because it can be used as a fuel to generate electricity and, once depleted, it can further be used for sowing, since the waste is still rich in minerals [12]. Microbial fuel cells (MFCs) are bioelectrochemical devices in which the chemical energy of the substrates is converted into electrical energy. There are many cell types, although in general the cell is connected to an external circuit; the composition of the cell consists of an anodic and cathodic chamber separated by a proton exchange membrane. The anodic chamber is customarily in anaerobiosis, and the cathodic chamber is in contact with oxygen [13,14,15]. In this sense, Cecconet et al. (2018) used agro-industrial waste in their cells, which were made with graphite electrodes, and managed to generate voltage peaks of 637 mV with an external resistance of 20.4 Ω on day 51. The excellent production of electricity obtained was attributed to colonization of the species of electroactive bacteria on the anode electrode [16]. In the same way, Asefi et al. (2019) used food waste as substrate and carbon felt as electrodes, and managed to generate voltage peaks of approximately 775 ± 2 mV and 422 mW/m^2^, with the decline in values being attributed to the lack of nutrients in the last days of monitoring [17]. Although vegetable residues are difficult to use for the generation of electrical currents, Fogg et al. (2015) were one of the first to report the use of plants in MFCs for electricity generation, pointing out their great potential for use as a substrate in a cell due to their possessing many active redox mediators [18]. In the same sense, Shrestha et al. (2016) managed to generate peak voltages of approximately 0.78 V and current densities of 1.504 A/cm^2^ in their MFCs [19]. These results were attributed to the high concentration of carbohydrates, amino acids, and species with redox activity [20]. Due to this, vegetable residues with high redox species and carbohydrate content are potentially excellent candidates for generating electrical current in MFCs when used as substrates. One of the most important agricultural products in the world is onion (*Allium cepa* L.), with 392,536 tons being produced in 2018, and its production continues to grow [21]. This product is a rich source of polyphenol dietary fibres and antioxidants, and onion skin is a waste that contains a high concentration of flavonoids compared to the edible part [22]. Flavanols have a higher concentration (280–400 mg/kg) in onions than in other vegetables; for example, broccoli has a concentration of 100 mg/kg and apple 50 mg/kg; thus, the onion contains active antimicrobial agents [23]. Due to the incredible popularity of this vegetable, large amounts of waste are being generated, which is used to flavour food products, especially dairy products such as flavoured cheese, sour cream, or meat products such as processed hams, cold cuts, and meat cans [24,25].

Worldwide, approximately 66 million tons of onions are produced each year. It is expected that in 2025, an increase of 88.6–92.8% of waste generated by this vegetable will occur, with the implementation of strategies to minimize contamination due to this product being of vital importance [26]. The accelerated increase in waste affects the environment, emphasising the need to design adequate strategies to minimize the social and environmental impacts on future generations [27]. In general, a 70% increase in the generation of urban solid waste is expected by 2050, which will occur if there are no changes in consumer habits in the process of disposing waste. The linear economy model follows a step-by-step purchase, manufacturing, and disposal scheme; this means raw materials are collected and used until they are finally discarded. This creates a domino effect where it results in a shortage of raw materials and increases in cost due to the handling and disposal of waste [28,29]. The circular economy and its acceptance have a direct effect on the fight against waste, since it uses waste as a product, contributing to the minimization of pollution [30]. To achieve positive changes with respect to the conservation of the environment, the use of innovative technologies for solid waste management must be counted on, taking the circular economy as the main focus, since it is framed around the valorisation of waste as a resource [31]. Countries and their citizens have the responsibility of producing structural changes in policies and plans related to the sustainable development of solid waste, achieving adequate treatment, management, utility, and final disposal through the circular economic model [32].

The main objective of this research is to generate electricity using onion waste (monitoring the physical-chemical parameters) and to identify the main microorganisms in anodic electrodes that generate such energy. In order to provide a clear picture, the voltage, current, pH, brix degrees, and conductivity were monitored for 35 days. The internal resistance, power density, current density, and the initial and final transmittance spectrum of the substrates were characterized by Fourier transform infrared spectroscopy (FTIR). Molecular techniques identified electrogenic bacteria attached to the anode. This research provides a solution by which the waste originating from this vegetable can be used as fuel and the eco-friendly generation of electricity, to the benefit of farmers and export and import companies.

## 2. Materials and Methods

### 2.1. Construction of Single-Chamber Microbial Fuel Cells

**Figure 1:**
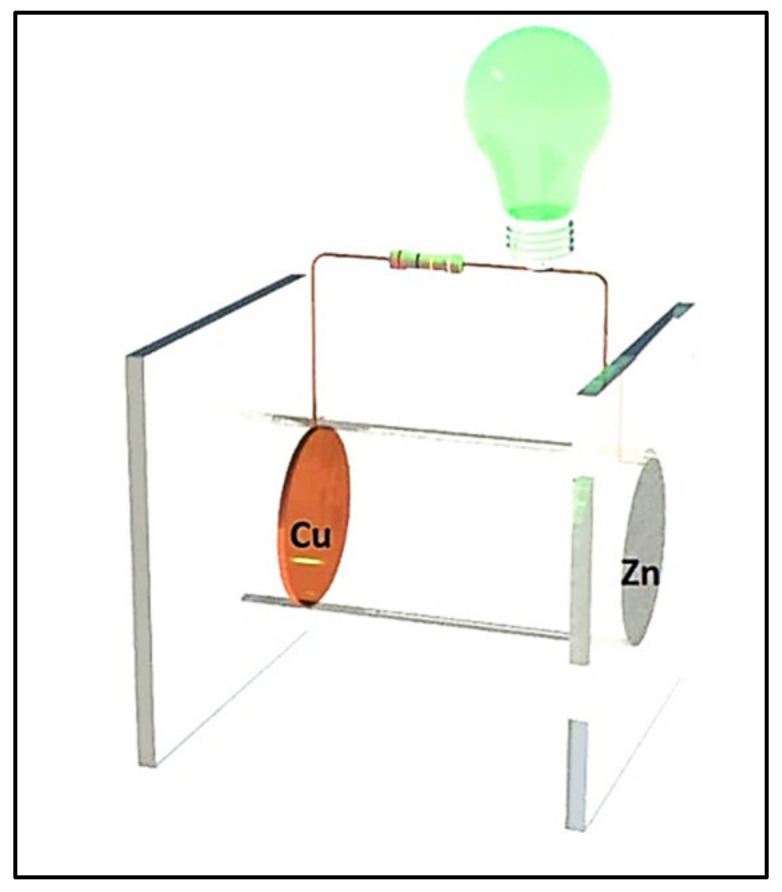
Scheme of the MFC prototype.

Microbial fuel cells (three in total) were created using copper (Cu) at the anode and zinc (Zn) at the cathode in the absence of a proton exchange membrane, as shown in the prototype in Figure 1. A 600 mL polymethylmethacrylate tube was used as the MFC chamber, in which a 5 cm hole was drilled at one end so that the cathode had contact with the environment (O_2_). The electrodes were 78.50 cm^2^ in area; both electrodes were joined by an external resistance connected with copper wire (0.2 cm in diameter).

### 2.2. Onion Waste Collection and Preparation

An amount of 3 kg of decomposed onion was collected from the La Hermelinda Trujillo market, Peru, in sealed airtight bags and stored in a cooler. The debris was placed in plastic trays to remove any plastic, paper, or other foreign material. The samples were then washed with distilled water three times to remove dust, insects, or other impurities. Next, the samples were left to dry in an oven (Labtron, LDO-B10) for 24 h 30 ± 1.5 °C. Finally, the onion residues were placed in an extractor (Maqorito-400 rpm) to obtain 800 mL (150 mL for each MFC) of waste onion juice.

### 2.3. Characterization of Microbial Fuel Cells

The voltage and current values generated were monitored using a multimeter (Prasek Premium PR-85-USA) for a period of 35 days with an external resistance of 1000 Ω at 22 ± 2 °C. Current density (CD) and power density (PD) were calculated using the equations of CD =$\;V_{cell}^{2}$/$R_{ext.}A$ and PD =$\;V_{cell}$/$R_{ext.}A$, where A (area) of the cathode has an approximate value of 78.50 cm^2^ [33]. With external resistances (Rext.) of 0.3 (±0.1), 0.6 (±0.18), 1 (±0.3), 1.5 (±0.31), 3 (±0.6), 10 (±1.3), 20 (±6.5), 50 (±8.7), 60 (±8.2), 100 (±9.3), 120 (±9.8), 220 (±13), 240 (±15.6), 330 (±20.3), 390 (±24.5), 460 (±23.1), 531 (±26.8), 700 (±40.5) and 1000 (±50.6) Ω [16]. Changes in conductivity (CD-4301 conductivity meter), pH (110Series Oakton pH meter) and Brix degrees (RHB-32 Brix refractometer) were also measured. Transmittance values were measured by FTIR (Thermo Scientific IS50) and MFC resistance values were measured using an energy sensor (Vernier- ±30 V and ±1000 mA). The data points of the voltage, current, pH, conductivity, ° Brix, Current density (CD) and power density (PD) figures represent the average values from three replicates and the error bars represent the corresponding standard deviations.

### 2.4. Isolation of Electrogenic Microorganisms in Anodic Chamber

For isolation, a swab of the anode plate was made; then, it was seeded by the stria technique in culture media such as Brain Heart Infusion Agar, Nutritive Agar, Mac Conkey Agar, and Sabouraud Agar. They were incubated at 36 °C to isolate Gram-negative bacteria and 30 °C for fungi and yeasts. The isolation of microorganisms was carried out in duplicate.

### 2.5. Molecular Identification of Bacteria and Fungi

The Laboratory’s Analysis and Research Center of “Biodes Laboratorios” carried out the molecular identification. From axenic cultures, they carried out DNA extraction using the CTAB technique. The MACROGEN Laboratory sequenced the PCR products, then analysed them by the MEGA X bioinformatics software (Molecular Evolutionary Genetics Analysis); finally, they were aligned and compared with the BLAST bioinformatics program (Basic Local Alignment Search Tool) to obtain the percentage of identity in identifying fungi and bacteria.

## 3. Results and Analysis

**Figure 2:**
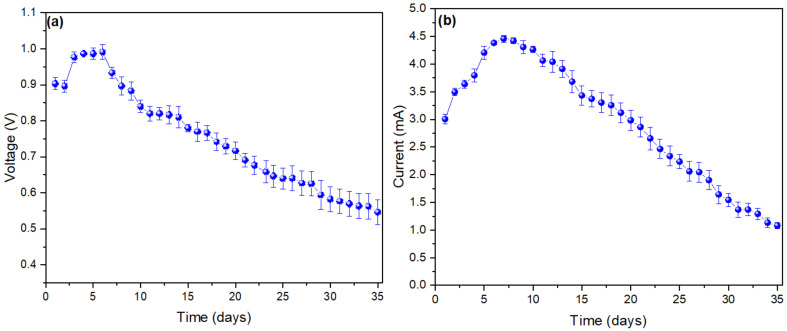
Monitoring of the values of (**a**) voltage and (**b**) current of the microbial fuel cells.

In Figure 2a, the voltage values observed during the 35 days of monitoring of the MFCs are shown, in which the successive increase of the values can be seen from the first (0.9033 ± 0.016 V) to the sixth day (0.991 ± 0.02), and then slowly decay until the last day (0.5463 ± 0.0345 V). The rapid generation of voltage in the first days is mainly due to the metallic electrode used. According to Hindatu et al. (2017), the use of an anodic electrode of this type has lower resistance and higher conductivity of the electrons generated to the cathode electrode [34]. The use of Zn and Cu as electrodes has already been studied; rapid generation of voltage on the first day was observed due to the current chemical present in the substrate, and because there was not enough time to adhere the microorganisms on the electrode [35,36]. However, as the days go by, the microorganisms exhibit increasing anodic behaviour due to the decomposition of the substrate [37]. At the same time, the voltage variations are generated by the substrate used, which contains components that affect the growth of microorganisms at the time of degradation [38]. Figure 2b shows the generated values of electric current during the 35 days of monitoring. It can be seen that, after the first day (3.006 ± 0.0837 mA), the current values increase to their maximum peak on the seventh day (4459 ± 0.0608 mA), following which a decrease is observed until the last day (10806 ± 0.0540 mA). The work carried out by Din et al. (2020), in which they used potato waste, mentions that the decrease in current values is due to the rapid hydrolysis of organic matter (substrate) due to the variation of the appropriate pH values [39].

On the other hand, glucose consumption by microorganisms under anaerobic conditions produces carbon dioxide, protons, and electrons in the anode chamber, which contributes to the increase in current values [40]. Likewise, the possible low resistance of the system (substrate, electrodes, and external circuit) contributes to making the flow of electrons more feasible, and the high current values during the monitoring period confirms the presence of an exoelectrogenic biofilm (community bacterially or electrochemically active) on the anode electrode [41,42]. These preliminary results on the use of onion waste for the generation of bioelectricity show the way for more research due to the excellent current and voltage results obtained, although as mentioned by Rahman et al. (2021), one of the essential points is the study of the availability of glucose by the substrates, which is what limits the release of electrons and leads to a decrease in electrical current [43].

**Figure 3:**
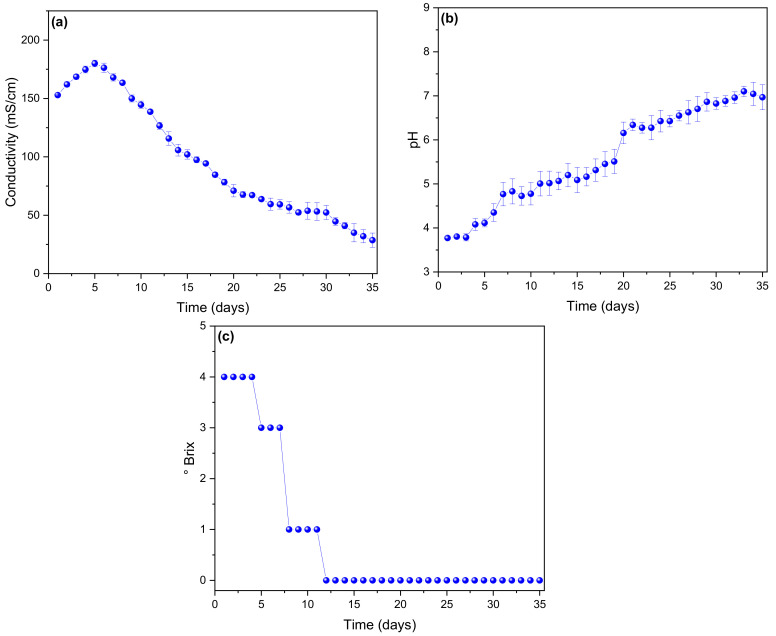
Values of (**a**) conductivity, (**b**) pH, and (**c**) ° Brix of the microbial fuel cells.

The maximum value of conductivity is obtained on the fifth day (179,987 ± 2859 mS/cm), as can be seen in Figure 3a, and conductivity then decreases continuously until the last day (28,667 ± 6110 mS/cm). The variations in conductivity are due to the creation and formation of sediments from the waste used during the electricity generation process [44]. While the increase in conductivity is mainly due to the reduction of the resistance of organic waste used as fuel, these values can be improved by adding inorganic salts to the substrate of the anode chamber [45,46]. In Figure 3b, it can be observed that the pH values vary from slightly acidic (3.77 ± 0.036) to neutral (6.968 ± 0.286), with the optimum pH for the generation of voltage and current being 4.35 ± 0.201. According to Geng et al. (2020), an MFC operating at neutral pH decreases the electrical parameters due to the competition between methanogens and electrigens contained in the substrate [47]. However, the optimal operation for each substrate and cell type varies due to the different operating parameters in electricity production [48]. For example, Ren et al. (2018) investigated the performance of their cells by adjusting the pH, managing to obtain the optimal pH for a value of 9, because the microorganisms present in their substrate obtained the appropriate conditions for their growth [49]. Figure 3c shows the ° Brix values exhibited by the cells during monitoring, which in the first days have a value of 4 and then drop to zero by the twelfth day, a value which is maintained until the last day of operation of the cells. Clark et al. 2018 [50] determined the presence of soluble solids (0.997, 0.1 ° Brix), pyruvate (0.825, 0.8 μmol g-1 FW), fructan (0.98, 1.9 mg g-1 FW), glucose (0.941, 1.1 mg g-1 FW), fructose (0.967, 1.0 mg g-1 FW) and sucrose (0.919, 1.7 mg g-1 FW) in onion crops by FTIR, as they are carbon sources for the growth of microorganisms.

**Figure 4:**
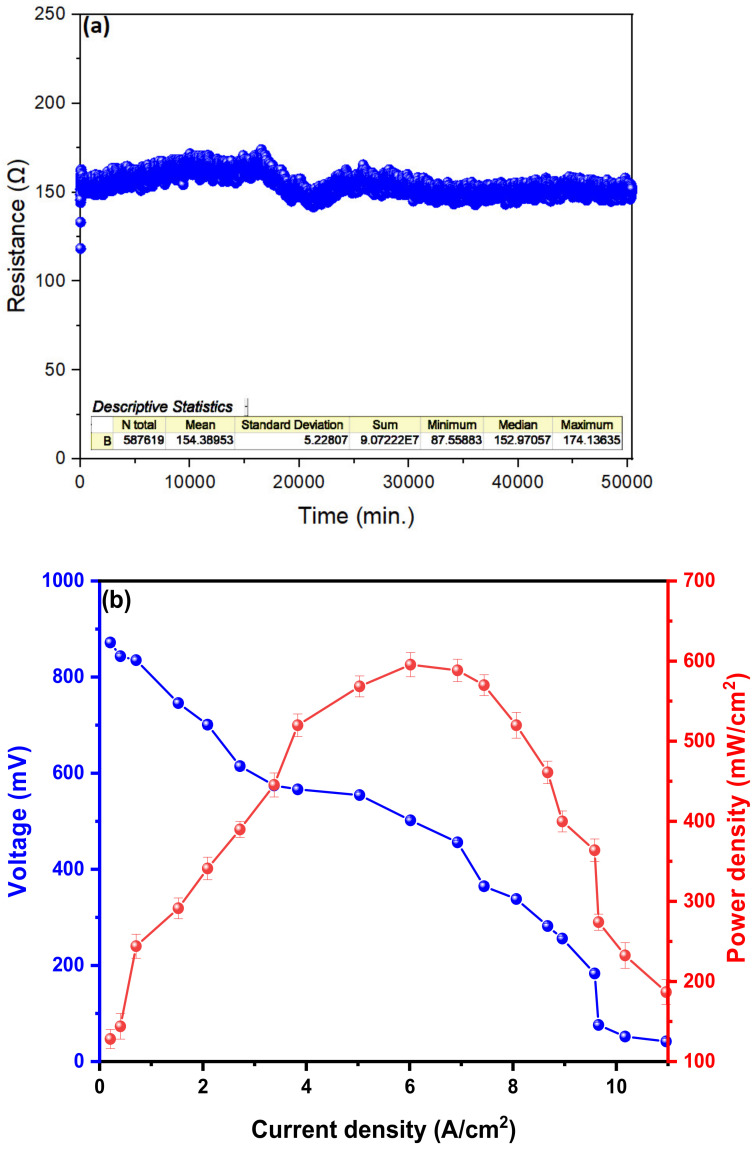
Characterization of (**a**) internal resistance and (**b**) power and voltage density about the current density of the MFCs.

Figure 4a shows the internal resistance values (Rint.) The resistance values of the microbial fuel cells during the 35 days (50,400 min) of monitoring did not show major fluctuations at 22 ± 2 °C, and the average value of Rint. Was 154,389 ± 5228 Ω. Previous studies show that low internal resistance is mainly due to good biofilm formation on the anode electrode due to electrogenic microorganisms present in the substrate [51,52]. In the same way, by containing a low Rint., electron transfer will occur more efficiently from anode to cathode; thus, microorganisms may prefer more direct ways of electron transfer due to their genetics [37]. Figure 4b shows the values of power density (PD) and voltage as a function of current density (CD), managing to generate a PD_max._ of 595.69 ± 15.05 mW/cm^2^ in a CD 6.02 A/cm^2^ and with a maximum voltage of 871.92 ± 7.9 mV. The PD and CD values obtained in this investigation were higher compared to those obtained with other substrates (*H. undatus*, *M. citrifolia* and *R. ulmifolius*) [53], using the same design and materials, which may be due to greater ease of degradation of the grape substrate [54]. In the same sense, in the work carried out by Yang et al. (2019), the values of CD and PD using sediments as substrate are lower than those obtained by us, thus demonstrating the great potential of fruit residues for the generation of electricity [55]. Although the CD and PD values can still be increased using a proton exchange membrane (MIP) between the anodic and cathode chamber, as demonstrated by Asensio et al. (2018) in his work, in which he used wastewater and different types of IPM as a substrate (Nafion-117 HRT3.16, Nafion-117 HRT 6.32d, Neosepta CMX, and Neosepta AMX), achieving a higher current density (~850 mA/m^2^) using the NAFION-117 HRT 3.16 as MIP [56].

**Figure 5:**
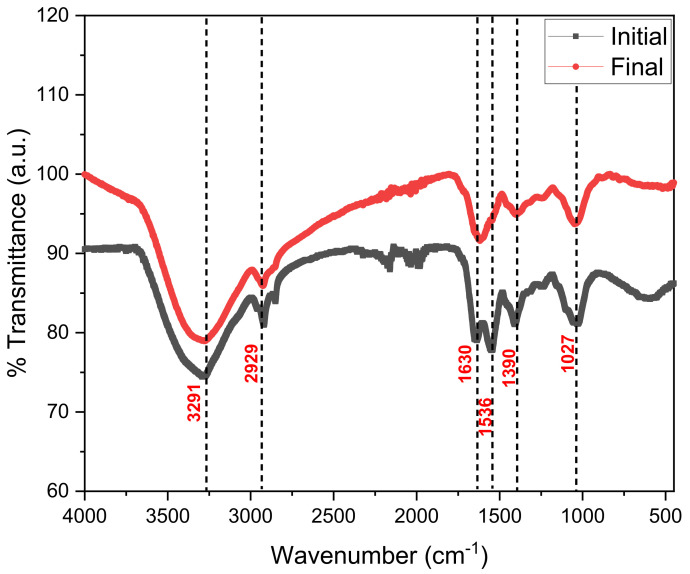
FTIR spectrophotometry of the initial and final onion residues.

Figure 5 shows the values of the transmittance spectrum by FTIR of the onion waste at the initial and final time of operation in the microbial fuel cells. The 3291 cm^−1^ peak belongs to the hydroxyl group (range 3200–3300 cm^−1^), the stretches close to 2929 cm^−1^ belong to the methylene-CH, the 1630 cm^−1^ peak belongs to the quinone or conjugated ketone (C=C, stretch), the peaks between 1300–1400 cm^−1^ belong to OH bonds and the 1027 cm^−1^ peak to -CC-stretch, ethers [57,58,59]. The decrease in the intensity of the transmittance peaks is mainly due to the degradation of the compounds in the electrical energy generation process [60].

The Analysis and Research Center of the “Biodes Laboratorios” laboratory carried out the molecular identification. The genetic material (DNA) was extracted from pure or axenic cultures of bacteria and fungi, isolated from anode plates with onion substrate, using the CTAB extraction method [61]. The PCR products for the 16S rDNA gene (bacteria) and the ITF region (yeast) of each isolate were sequenced in the Macrogen laboratory (USA) [62]. Then the relationship of the sequences was evaluated in the bioinformatics Software MEGA X (Molecular Evolutionary Genetics Analysis) to be later aligned and compared with other sequences in the bioinformatics program BLAST (Basic Local Alignment Search Tool), through which the percentage of identity was obtained for the identification of bacteria and fungi.

A BLAST characterization of the rDNA sequence of the bacteria and yeast isolated from the anode plate of the onion microbial fuel cells was performed, where a 100% identity percentage was obtained corresponding to the Pseudomona eruginosa species, 99.93% to the Acinetobacter bereziniae species, 100%, to the Stenotrophomonas maltophilia species (see Table 1), and 100% to the Yarrowia lipolytica species (see Table 2).

**Figure 6:**
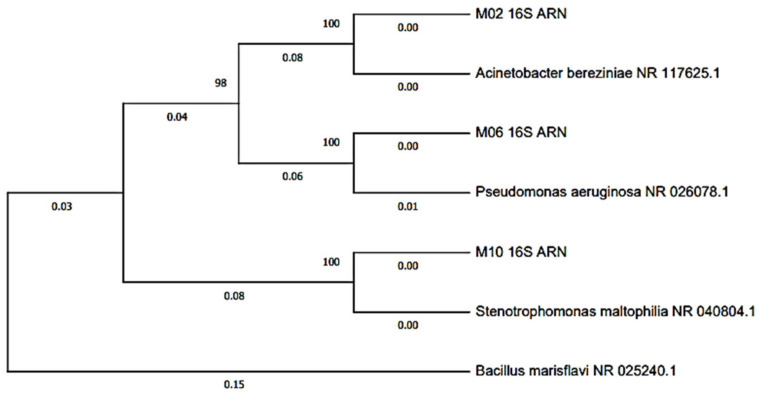
Dendrogram of groups of bacteria isolated from the MFC anode plate with onion substrate.

The phylogenetic tree was constructed using the Maximum Likelihood method with Bootstrap phylogeny test with 100 replicates to show differences in general phylogenetic distances. A BLAST characterization of the rDNA sequence of the bacteria isolated from the anode plate of the fuel cells was performed (Figure 6). Onion microbial bacteria, identified as *Pseudomonas aeruginosa* species, is a Gram-negative, facultative aerobic bacterium [62], which can use carbon and nitrogen sources, obtaining energy from the oxidation of sugars; this species is persistent in the environment [63]. Likewise, it is worth mentioning that this species possesses electron mediators such as phenazine-1-carboxylic acid, pyocyanin, pyoverdine, among others, which allows it to survive in anaerobic conditions [64,65].

A study by Ali et al. exposed the efficiency of using the species Pseudomonas eruginosa, which generated 136 ± 87 mW/m^2^ from the use of glucose, followed by fructose and sucrose. This study observed that the cell fed with glucose presented higher bacterial adhesion [66]. On the other hand, *Acinetobacter bereziniae* was identified as a Gram-negative, aerobic, non-fermentative, oxidase-negative, and immobile organism [67]. This microorganism has been detected as a contaminant in human and animal milk and the environment. This bacterium is characterised by surface hydrophobicity, which contributes to its adherence to surfaces [68]. Likewise, this genus was proposed as a model microorganism for environmental microbiological studies, pathogenicity tests, and industrial chemical production [69].

**Figure 7:**
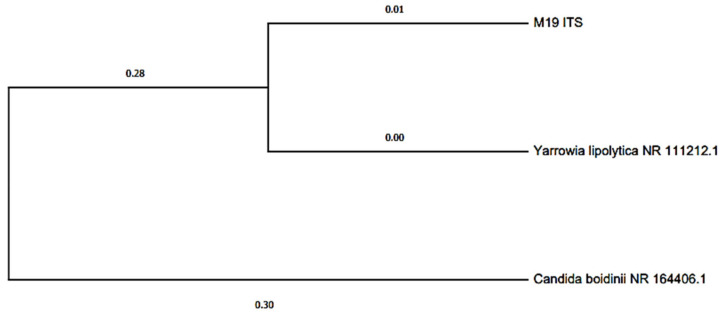
Dendrogram based on the ITS regions of the rDNA regions of a *Yarrowia lipolytica* culture isolated from the anode plate of the MFC with onion substrate.

It is worth mentioning that the species Stenotrophomonas maltophilia was also identified, a cosmopolitan and ubiquitous bacterium found in a series of environmental habitats, mainly associated with plants [70]. This bacterium can form biofilms on various surfaces [71]. These bacteria transfer electrons directly to the anode through outer membrane carrier proteins, such as cytochrome c, or membrane appendages called nanowires [72]. Romo et al. 2019 investigated the composition of the microbial community of the cathode of a Microbial Fuel Cell for the reduction of Cr (VI) in tannery effluents, finding four bacterial phyla (Proteobacteria, Actinobacteria, Fimicutes and Bacteroides) by pyrosequencing 454 of the 16S rRNA gene, with the genus Pseudomonas being the most abundant. On the other hand, they stressed the importance of the biofilms formed in the electrodes and the generation of electricity through microbial combustion cells with a salt bridge. In this way, the system’s potential is demonstrated together with the microbial communities in the bioremediation processes of contaminated effluents [73]. The phylogenetic tree for yeast was built with the Maximum Likelihood method without Bootstrap phylogeny test, for which ribosomal DNA sequences based on the ITS regions of the *Yarrowia lipolytica* species were used (Figure 7). It is an ascomycete yeast with a high lipolytic and proteolytic capacity. This species has been isolated from meat, fermented dairy, sewage, and water contaminated by hydrocarbons [74]. In Santiago et al. 2020, a *Sacharomyces cereviceae* culture was used as fuel, generating a voltage of 0.761 volts and a PD_max_ and CD_max_ of 8196 mW/cm^2^ and 8383 mA/cm^2^, respectively, in the Zn-Cu cell, while in the Zn-Zn cell, 5684 mW/cm^2^ and 0.238 mA/cm^2^ of PD_max_ and CD_max_ were generated, respectively.

## 4. Conclusions and Future Development

Bioelectricity was successfully generated using onion waste using low-cost microbial fuel cells manufactured with zinc and copper electrodes. The maximum voltage and current obtained during the monitoring were 0.991 ± 0.02 V and 4.459 ± 0.0608 mA, respectively, values which, compared to other reports in the literature, are higher and show suitable electrical parameters that did not decrease in their entirety by the end of the monitoring. The optimal pH in the cells was 6.968 ± 0.286, and its conductivity values increased to 179.987 ± 2.859 mS/cm, while the ° Brix rapidly dropped to zero on day 12. The internal resistance of the cells was 154.389 ± 5.228 Ω, which explains why, of its high electrical values, this low resistance value is mainly due to the compounds of onion residues, which decrease due to degradation in the bioelectricity generation process, as shown by the transmittance spectrum of the FTIR. The maximum power density was 595.69 ± 15.05 mW/cm^2^ at a current density of 6.02 A/cm^2^ with a maximum voltage of 871.92 ± 7.9 mV. Finally, a microbial consortium adhered to the anode was identified by molecular techniques, which obtained a percentage of identity of 100% to the *Pseudomona eruginosa* species, 99.93% to the *Acinetobacter bereziniae* species, 100% to the *Stenotrophomonas maltophilia* species, and 100% to the species *Yarrowia lipolytica*.

In the near future, researchers will face new challenges transitioning from the laboratory to real social environments such as installations of CFMs in houses, industries and food supply centres, the passivation of electrode materials by electromechanical deposition to improve the effects of corrosion of the material, and the connection of the electrodes in the MFCs. Likewise, the effects that can be given by using different nanomaterials for coating the electrodes should be investigated.

## Figures and Tables

**Table 1:** BLAST characterization of the rDNA sequence of bacteria isolated from the MFC anode plate with onion substrate.

BLAST Characterization	Consensus Sequence Length (nt)	% Maximum Identity	Accession Number	Phylogeny
*Pseudomona aeruginosa*	1442	100.00%	MT633047.1	Cellular organisms; Bacteria; Proteobacteria; Gammaproteobacteria; Pseudomonadales; Pseudomonadaceae; Pseudomonas; Pseudomonas aeruginosa group
*Acinetobacter bereziniae*	1468	99.93 %	CP018259.1	Cellular organisms; Bacteria; Proteobacteria; Gammaproteobacteria; Pseudomonadales; Moraxellaceae; Acinetobacter
*Stenotrophomonas maltophilia*	1477	100.00%	NR_041577.1	Cellular organisms; Bacteria; Proteobacteria; Gammaproteobacteria; Xanthomonadales; Xanthomonadaceae; Stenotrophomonas; Stenotrophomonas maltophilia group

**Table 2:** BLAST characterization of the yeast rDNA sequence isolated from the MFC anode plate with onion substrate.

Caracterización BLAST	Consensus Sequence Length (nt)	% Maximum Identity	Accession Number	Phylogeny
*Yarrowia lipolytica*	369	100.00%	MN124085.1	Cellular organisms; Eukaryota; Opisthokonta; Fungi; Dikarya; Ascomycota; saccharomyceta; Saccharomycotina; Saccharomycetes; Saccharomycetales; Dipodascaceae; Yarrowia

## Data Availability

Not applicable.
